# Co-development of client involvement in health and social care services: examining modes of interaction

**DOI:** 10.1108/JHOM-10-2022-0310

**Published:** 2024-02-12

**Authors:** Anna-Leena Kurki, Elina Weiste, Hanna Toiviainen, Sari Käpykangas, Hilkka Ylisassi

**Affiliations:** Finnish Institute of Occupational Health, Helsinki, Finland; Tampere University, Tampere, Finland; Finnish Institute of Occupational Health, Tampere, Finland; Finnish Institute of Occupational Health, Oulu, Finland

**Keywords:** Activity theory, Client involvement, Co-development, Co-configuration, Interaction, Social services, Health organization

## Abstract

**Purpose:**

The involvement of clients in service encounters and service development has become a central principle for contemporary health and social care organizations. However, in day-to-day work settings, the shift toward client involvement is still in progress. We examined how health and social care professionals, together with clients and managers, co-develop their conceptions of client involvement and search for practical ways in which to implement these in organizational service processes.

**Design/methodology/approach:**

The empirical case of this study was a developmental intervention, the client involvement workshop, conducted in a Finnish municipal social and welfare center. The cultural-historical activity theory (CHAT) framework was used to analyze the development of client involvement ideas and the modes of interaction during the intervention.

**Findings:**

Analysis of the collective discussion revealed that the conceptions of client involvement developed through two interconnected object-orientations: Enabling client involvement in service encounters and promoting client involvement in the service system. The predominant mode of interaction in the collective discussion was that of “coordination.” The clients' perspective and contributions were central aspects in the turning points from coordination to cooperation; professionals crossed organizational boundaries, and together with clients, constructed a new client involvement-based object. This suggests that client participation plays an important role in the development of services.

**Originality/value:**

The CHAT-based examination of the modes of interaction clarifies the potential of co-developing client-involvement-based services and highlights the importance of clients' participation in co-development.

## 1. Introduction

A current trend in the development of the working practices in health and social care is to involve clients in the development, evaluation and production of services alongside professionals and managers (e.g. Nykänen *et al.*, 2022; Viksveen *et al.*, 2022; Weiste *et al.*, 2022; Yang *et al.*, 2019). This means that giving clients an active role and a say in their services is a central value (Nykänen *et al.*, 2022; Sihvo *et al.*, 2018; Viksveen *et al.*, 2022). When clients are actively involved in their care, several positive outcomes can be achieved, including improved continuity of care, better health outcomes, increased client satisfaction and reduced healthcare costs (Viksveen *et al.*, 2022; Yang *et al.*, 2019).

Client involvement, i.e. giving clients a choice, voice and co-productive role in the provision of services, has required renewing of client–professional relationship (Nykänen *et al.*, 2022). Whereas clients have traditionally been regarded as objects of treatment, today they are considered to have the right to express their opinion and to be involved in the decision-making concerning the services they use (Sihvo *et al.*, 2018; Viksveen *et al.*, 2022; Yang *et al.*, 2019). Prior research has found a gap between the ideal and practice (e.g. Lord and Gale, 2014; Weiste *et al.*, 2022; Yang *et al.*, 2019; Viksveen *et al.*, 2022). Professionals appreciate client involvement, but they may reproduce the institutionally bounded roles with power asymmetries even unconsciously (Nykänen *et al.*, 2022). Some professionals feel equalized power relations with active clients are a threat to professional competencies and are hesitant to adopt client involvement as their guiding work practice (Anthony and Crawford, 2000; Viksveen *et al.*, 2022; Weiste *et al.*, 2020) In addition, customary work practices, organizational structures and divergent visions of the contents of services may also hinder the adoption of the new client involvement-based orientation (Engeström and Kerosuo, 2003; Lord and Gale, 2014).

In recent decades, the principles of client involvement have been implemented in Finland through legislation (e.g. Act on the Status and Rights of Patients 785/1992 [1]; Act on the Status and Rights of Social Welfare Clients 812/2000 [2]) and several public sector reforms (Kaatrakoski, 2016). Two partially contemporaneous models that apply the principles of private sector management have led to an increasing demand for client involvement. The first model, New Public Management (NPM), perceives clients as “consumers” of public services. Traditionally, the NPM model has emphasized the resource constraints and adopted a managerial approach to delivery of services and allocation of scarce resources (Osborne and Strokosch, 2013; Kaatrakoski, 2016). This has led to a product-driven conception of services where co-production excludes client involvement in the process of planning and developing services (Grönroos, 2011; Osborne, 2018). The second model, public service-dominant logic, emphasizes interaction between the service producer and service user when producing new services. The interdependency between service producer and client is appreciated on the operational level (Osborne and Strokosch, 2013; also, Boaz *et al.*, 2016). Thus, the value of the client and the public service organization is created at the nexus of the interaction in the context of the client’s wider life experience (Grönroos, 2011; Osborne, 2018).

This study applies the cultural-historical activity theory (CHAT) perspective to the joint development of services. The quest for client orientation and joint development has the background in the historical transformation of work and emerging modes of knowledge and production (e.g. Engeström, 2004; Kaatrakoski, 2016; Nummijoki, 2020). Mass production has given way to the forms of co-configuration, in which producers and users are increasingly coupled in collaborative endeavors to develop services that meet the client needs (Daniels *et al.*, 2019; Engeström, 2004; Victor and Boynton, 1998).

We posit that client involvement is an essential element in the co-configuration of health and social care services. By applying the CHAT approach, we explore how professionals and managers, together with clients, jointly develop their conceptions of client involvement and search for practical ways in which to implement them in service processes. We refer to their co-configurational activity in the workshop by the term “co-development” (Virkkunen and Schaupp, 2011). The study examines, firstly, what *object-orientations* to client involvement and service production the professionals and clients bring to the discussion. Secondly, the study analyses the transitions in *the modes of interaction* from minimal “coordination” to productive “cooperation” and “reflexive communication” (Engeström, 2008; Fichtner, 1984) furthering the co-development. The CHAT-based analytical concepts are explained in the next section.

## 2. Cultural-historical activity theory (CHAT) as the framework

Cultural-historical activity theory has been developed during the past decades as an approach to the challenges of change and learning in collective work practices (Daniels *et al.*, 2010). In the healthcare settings, the CHAT framework lends itself to the complexity of interacting systems involving multiple activities, not only in and across medical care providers but also health and social services, education and the clients’ life. The interventionist methodology allows to combine research and development projects aiming to transform the healthcare practices or education, or both (Engeström and Pyörälä, 2021).

The CHAT-based framework has informed the analysis of shared decision-making between client and provider (Witkop *et al.*, 2021) and helped in addressing complex healthcare problems across diverse settings and levels of policy, management and clinical care (Greig *et al.*, 2012). CHAT is applied to organizational transformation of healthcare systems and to the implementation of tools and practices in different sectors (Kirk and Andersen, 2019). In medical education, the interventionist methodology has been applied to develop and research the often-problematic transitions between medical school and workplaces that provide the real-life clinical learning environments and placement after graduation (Reid *et al.*, 2015, Skipper *et al.*, 2021).

Common to the studies is focusing on the collective *object of activity* that is inherently contradictory and can be contested by collaborating parties and stakeholders of care. This is due to the multiplicity of participant activities stemming from their cultural and historical backgrounds, which is observable in different object-orientations between, for example, client care and training, or nursing, medicine and social service. Basically, the object of healthcare can be defined as by Engeström and Pyörälä (2021, p. 8):

An activity system is oriented toward an object. The object embodies the long-term purpose of the activity, generating horizons for possible actions. In healthcare, the general object is health and illness, whereas each specific patient with a specific complaint is a situated manifestation of the object.

Client involvement can be defined as a significant change in the object of activity, referring to both the contents of the services and the way in which the services are produced (Engeström, 2015; Toiviainen and Weiste, 2022). Client involvement expands the object, allowing multiple voices to participate in and contribute to the creation of good care. We use the term “object-orientation” to characterize the perspectives though which the new object of activity is constructed.

The co-development for the expansion of the object depends on the quality of interaction among the participants. *The modes of interaction* distinguish different levels of collaboration, defined as “coordination”, “cooperation” and “communication” (Raeithel, 1983; Fichtner, 1984). Fichtner (1984) claimed that traditional learning and instruction theories tend to separate cooperation and communication as forms of social interaction and instruction techniques, on the one hand, from the contents of learning, on the other. The reflexive relation between the *content* and *forms of social interaction* is important for co-development activity. Here, the content of interaction refers to the object of activity to be co-developed, i.e. the client involvement.

By analyzing collaboration and learning in working-life teams, Engeström (2008) further developed three modes of interaction (Table 1). Each mode of interaction sets a different scene for the aspired expansion of the object of activity. In this framework, the regulation of the activities of the multiple actors and the creation of a shared object is believed to take place according to a tacit or explicit script of collaboration (Seppänen and Toiviainen, 2017) specific to each mode of interaction (see Table 1).

Activity theoretical research posits that the transitions between modes of interaction can be analyzed through specific turning points (Kärkkäinen, 1999; Toiviainen, 2003; Vetoshkina and Toiviainen, 2022). A turning point can be operationalized as a moment in discussion that either widens or narrows the conceptualization of the object in the discussion that follows (Toiviainen, 2003). An *expansive turning point* entails the collaborative reframing and expansion of the object and moving from the coordination to the cooperation or communication mode of co-development interaction (Engeström, 2008).

To summarize, there is a need for further research of how the health and social care professionals, managers and clients co-develop client involvement in view of future requirements of care. Co-development proceeds through identifying and solving client involvement problems during a developmental process and thus constructing the shared client involvement-based object of work. The research questions are:

RQ1. What object-orientations to client involvement do the participants co-develop?

RQ2. What are the expansive turning points in the modes of interaction from coordination to cooperation and reflexive communication when constructing client involvement?

## 3. Data and methods

### 3.1 The case: Client involvement workshop process

The data were drawn from a Client Involvement Workshop held in 2019 in a municipal health and welfare center that was engaging professionals and clients as active agents in a development project. The health and welfare center is located in a large city in Finland. The Client Involvement Workshop focused on developing services for frequent, regular users of *multiple* services: for example, psychiatric and substance abuse services. The aim was to offer solutions for fragmentation of care, which is a major challenge for healthcare practice (Engeström and Pyörälä, 2021).

**Figure 1:**
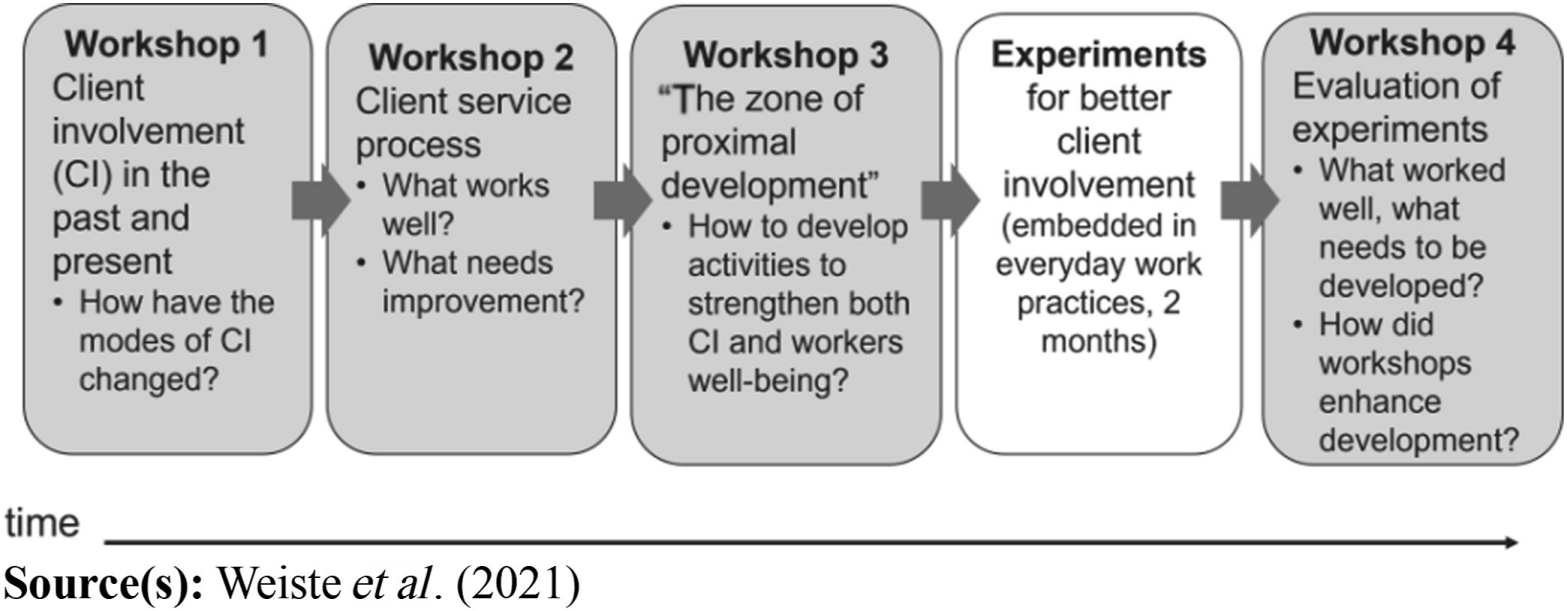
Client involvement workshop process

The Client Involvement Workshop process (Weiste *et al.*, 2021) applied the activity theory-based methodology, designed to enhance collective development and learning in major changes of activity (Engeström, 2015; Virkkunen and Newnham, 2013). It consisted of four sessions, conducted over a period of eight months, in which clients and professionals of different services developed the practices of client involvement and the fluency of client processes (Figure 1.).

Altogether 20 participants (8–12 in each session) and three researcher–facilitators (first and second author and their colleague) attended the workshop. The participants were 16 employees, two clients and two managers. The employees were nurses, social workers, physiotherapists, development specialists and department managers from four different service units, including the medical center, dental health, social work and psychiatry/substance abuse units. Two top managers from the medical center and social work unit took part in one of the sessions. The employees recruited clients to the workshops. They contacted clients they knew based on their previous participation in the development of services. Two clients answered positively: one was a trained expert-by-experience who worked part time as a peer provider (Noorani, 2013). The other client had already participated in another co-development group. The researchers guided the process to help the participants question and expand their ideas on client involvement, prospects and practical solutions (Virkkunen and Newnham, 2013).

Ethical pre-evaluation and institutional permission to use the data for research purposes were obtained from the Finnish Institute of Occupational Health Ethics Committee (23 November 2018). Written, informed consent was obtained from all the workshop participants. The anonymity of the participants was ensured by altering the details that may enable their identification in the text and data excerpts.

### 3.2 Method of analysis

The data consisted of audio-recorded and transcribed workshop discussions (12 h of interaction in total). First, we conducted a data-driven content analysis of the transcripts by separating thematic episodes dealing with “client involvement”, the general object of activity. In total, 267 thematic episodes were identified consisting of 1–147 speaking turns (on average 10 speaking turns). Meeting-technical themes (79 episodes) and the “other themes” category (13 episodes) were excluded from this analysis. The next phase was to specify and name the discussion topics of each episode. A topic is object-related; it refers to the participants' orientation to client involvement. The third phase was to answer RQ1 by clustering the object-related topics (175 episodes) into main categories. This step produced two categories of object-orientation (see Results): (1) Enabling client involvement in service encounters (113 episodes) and (2) promoting client involvement in the service system (62 episodes).

The fourth phase of analysis aimed to answer RQ2 regarding the expansive turning points from coordination to cooperation and reflexive communication. We defined the mode of interaction in each episode by analyzing the object and script of discussion (Table 1) and examined who or what generated the turning point indicating transition from one mode to another and constituted a contentual object-oriented change in the discussion.

## 4. Results

We found that client involvement was constructed through two interconnected object-orientations. In Section 4.1. we present these object-orientations named *Enabling client involvement in service encounters* and *Promoting client involvement in the service system.* In Section 4.2. we present our analysis of expansive turning points. We demonstrate that the coordination was predominant mode of interactions during the workshop discussions and illustrate the mechanisms through which the transitions from coordination to cooperation emerged. We show that the clients' perspective and contributions helped professionals to cross the organizational boundaries, and together with clients, construct a client involvement-based object.

### 4.1 Two object-orientations to “client involvement”

The two object-orientations *Enabling client involvement in service encounters* and *Promoting client involvement in the service system* *and* the topics through which these object-orientations are manifested during the developmental process are presented in Table 2.

In the first object-orientation, *Enabling client involvement in service encounters*, the participants highlighted that clients are expected to be involved in their own service in decision-making, identifying their needs, setting goals and planning and implementing the service. Central topics in this object-orientation were the clients' needs and their opportunities to *“be heard”* during their service encounters. Through the topics setting a goal, planning the service and putting the services into practice participants discussed the clients’ possibilities to be involved in own care and affect the solutions related to it. This was enabled though decision-making in client–professional interaction and collaboration between services during the service encounters.

In the second object-orientation, *Promoting client involvement in the service system*, the participants expected client involvement to be promoted through managerial and organizational practices, service structures and involving clients in development. Participants constructed these topics by discussing, for example, handling the customer feedback, the practices involving clients in service development, the involvement strategy of the city and the *“scorecard”* as an instrument to monitor client involvement. The discussions also drew connections between client involvement, productivity, effectiveness and work-related well-being. A developmental experiment, a new multi-professional consultancy practice that was expected to reduce the *“bouncing of the client from service to service”* was also an attempt to promote client involvement through organizational practices.

As Table 2 shows, the clients mainly contributed to the first object-orientation, *Enabling client involvement in service encounters* and to a lesser extent to the second one, *Promoting client involvement in the service system.* They described their personal experiences of service encounters and their viewpoints on service processes. In the following, we call these two object-orientations “Object-orientation 1” and “Object-orientation 2”.

### 4.2 Expansive turning points indicating change in mode of interaction

We found that coordination was the predominant mode of interaction and observed no reflexive communication in the discussion data. Therefore, we focused our analysis on the turning points that indicated transitions from coordination to cooperation. We identified 31 turning points, of which 21 were related to Object-orientation 1, *Enabling client involvement in service encounters* *and* ten to Object-orientation 2, *Promoting client involvement in service system*. To show how object construction emerged through modes of interaction, we first offer an example of coordination. We then present descriptions of the turning points from coordination to cooperation and provide examples of the two object-orientations.

#### 4.2.1 Coordination as a predominant mode of interaction

In coordination, each participant focused on a topic of discussion from their own point of view and on their own service. Excerpt below comes from the first workshop session, which discussed the theme of client involvement in the past and present. In the excerpt, the coordination is related to Object-orientation 1. The client and the professionals are discussing client needs, especially the needs of a client who frequently uses the services.

##### Excerpt 1 (session 1)

Client 1: I’m really navel gazing, but as an expert-by-experience, I can say that they want to hear a real person, a person who has experienced these things … that everything is possible, and that is what supports them. I’m basically the link between the client and the professional and I think that’s really important.

Interventionist 1: And your group, what did you discuss?

Professional 1: From my perspective, it’s important to get an appointment as soon as possible, that’s been the greatest need for ages.

Interventionist 1: Yes …

Professional 2: Well, every one of us is looking at it from our own perspective, so, in the health care center the need is to get a doctor's appointment as soon as possible … But then if we look at it more closely, we can see other alternatives, before ending up at the doctor's … some people contact us … they tell us straight out that it’s nice to have a chat [with the nurse] when they feel lonely

Interventionist 1: So, one central issue behind [these calls] is loneliness …

Professional 3: Well then, I’m from Youth Social Services, more adolescents are homeless than ever before, and they have mental health problems and/or addictive behavior, money problems, a need for health services.

Interventionist 1: Yes, so they need multiple services.

Client 1: We also had the mental ill-health of youth.

Professional 3: Yep, everything’s intertwined with it … clients with immigrant backgrounds have increased, and those from other parts of Finland too … and they are, or end up, homeless.

In the excerpt, all participants concentrate on their own role-based viewpoints. Client 1 starts the discussion by emphasizing the importance of experimental knowledge, peer support and the role of expert-by-experience, and Professional 2 continues by stressing that getting an appointment is a central need, both in the past and present. Professional 2 turns the discussion transiently to clients' expectations and interpretations of their service needs. However, during the same speaking turn they also address how service needs are discussed in the health center during the service encounter, thus, returning to the perspective of their own service. The discussion on service needs from the client’s perspective ends, and Professional 3 directs the discussion toward the professionals' perspective of social work. Thus, despite partially sharing the topic they fail to reach a shared object-orientation.

#### 4.2.2 The object-orientation 1: the turning points from coordination to cooperation

The turning points from coordination to cooperation occurred when the professionals rose above the interests of their “own” service and professional boundaries and the discussion shifted to the perspective of the client. The participants started to build a shared view of how client involvement is realized in service encounters and the factors that either hinder or foster it.

The most general type of turning point in object-orientation 1, *Enabling client involvement in service encounters* occurred when the client expressed or the professional explicitly asked for their viewpoint, which ruptured the otherwise somewhat general discussion. This is exemplified in Excerpt 2.

##### Excerpt 2 (session 1)

Interventionist 2: … and on the third level … how clients are also involved in influencing the service system.

Professional 3: … we must offer situations in which important and influential people and clients can meet, so that clients can directly tell them [their thoughts] and be heard.

[turning point 1, coordination ends, cooperation starts]

Client 1: And we have to remember that we [clients] are individuals with individual needs …

Interventionist 2: Yes. Isn’t this, like exactly the one part of client involvement, that those individual [Client 1 interrupts]?

Client 1: Yes, that’s the moment that determines everything.

[…]

Professional 3: Yes, yes … when you think that the professionals should also have resources for that, that is, have enough time. But if you have too much going on, the encounter isn’t going to be very good

Client 1: It’s really about the first encounter. What I hear daily out there is that the way in which they are received and so on, whatever the way, they don’t come back.

Professional 3: Yes. Our young clients, they turn on their heels very quickly.

Client 1: It’s challenging, and it takes a long time. Because already when they come for the first time, the work they have done beforehand, is huge. And that’s what we don’t really always remember.

Interventionist 2: Is it also a question of trust?

Client 1: Trust.

Professional 3: It really is. Sometimes it takes six months to build it. Or even longer.

Professional 9: And the first encounter. The interaction and dialogue in the situation can be extremely difficult.

Professional 3: Yes, and it’s not necessary the actual appointment, but before it, what they encounter in this huge building, at reception.

[turning point 2. cooperation ends, coordination starts]

Professional 1: Yes, I also take appointments at the dental center, and then I’m alone at the counter so if there’s a long queue of people already ….

In the excerpt, the turning point from coordination to cooperation occurs when Client 1 interrupts the general discussion on client involvement in the service system by highlighting the fact that clients are individuals with individual needs. The client and professionals discuss the potential and the challenges of the client, leaving the professional viewpoint to take a backseat. They start to construct a shared view on client involvement and discuss the importance of interaction, dialog and trust-building when involving clients in service encounters, but also in all the encounters at reception and in the corridors of the health and welfare center. However, this promising co-construction ends with one professional’s comments on appointment scheduling from her own point of view and own service and the interaction turns back to the coordination mode.

As Excerpt 2 shows, the turning points from coordination to cooperation occurred when the clients challenged the professionals, raising their own experiences and turning the discussion to the client’s perspective instead of that of the services. We found four additional types of turning points in Object-orientation 1: when the discussion turned to problematic client situations through the mirror data offered by the interventionist; when the professional took up a concrete client situation or otherwise turned the discussion to the client perspective; when the professional questioned, or expressed tensions in the current mode of activity; and when the professional questioned either the interventionist–facilitator or the assignment used in the workshop. In all the cooperation episodes (apart from the episodes arising through the mirror data) in our first object-orientation, the clients actively took part in the interaction.

We also found that in terms of content, Object-orientation 1 developed during the cooperation episodes. Excerpt 2 highlights the importance of interaction, dialog and trust-building when involving clients in service encounters. The clients' involvement in decision-making and its situational nature, as well as their involvement in their own medical data, were also central topics in the cooperation episodes through which this object-orientation was constructed.

#### 4.2.3 The object-orientation 2: the turning points from coordination to cooperation

The second example of a turning point from coordination to cooperation is related to Object-orientation 2, *Promoting client involvement in the service system.* Excerpt 3 below is from a group discussion in which the professionals refined a developmental experiment, a new consultancy practice. It exemplifies the type of turning point in which the professional questions the current mode of activity and highlights the tensions between the planned experiment and the current mode of activity. The group in question had no clients.

##### Excerpt 3 (session 3)

[cooperation starts after the crosstalk]

Professional 7: Did you say, or who was it, that it’s also question of supervisors' management, how we see that they really have time, and can an employee go and take part [in consultancies] … it also requires some kind of culture change and new procedures … it won’t work, if it [a consultancy] they say, yes, in a month's time. It should be possible now … or in half an hour.

Professional 2: … if it was possible to have a public health nurse in the room at that moment, or a nurse. On the fourth floor, we have a so-called queue nurse, could they be consulted … we don’t have this practice.

Professional 7: No, exactly, we just don’t have it. Or in general … asking, is it ok if the public health nurse comes here too. Or something like that, we could lower the thresholds and gain trust, and afterwards, the client would dare come to your floor.

Professional 2: … could this be implemented in such a way that it’s like, the [queue nurse] nurses' patients, other than those in the queue.

[ …. ]

Professional 7: We’re thinking here about the client experience or client involvement. But at the same we’re talking about productivity and effectiveness.

Professional 8: And the well-being of the employees.

Professional 2: Let me summarize, we’ve had a rich discussion, and we’re going to make the idea of a multi-professional consultancy something concrete, and our arguments are that it would mean less bouncing of the client from service to service, and when it starts working smoothly, the client feels involved. And then the employees also feel that their day-to-day work flows more smoothly.

The turning point occurred when Professional 7, during their turn, highlighted the tension between the experiment and the current way of organizing. They were worried that employees might not currently have time to take part in consultancies when needed. During this turn, Professional 2 and Professional 7 began to outline flexible working practices that could help the implementation of a new consultancy practice and started to sketch opportunities to develop the role of the so-called queue nurse. As Professionals 7, 8 and 2 elaborated at the end of the excerpt, the basis of a developmental experiment is to develop client involvement, but a new practice could also improve the workflow of the professionals and thus productivity, effectiveness and work-related well-being.

We also found two other types of turning points related to Object-orientation 2: the professional returning to the client’s earlier viewpoint and starting to reflect on it from the perspective of service structures; and the assignment given by the interventionist leading the professionals to conduct service development. During the cooperation episodes, the participants pondered how current service structures enable or restrict client involvement and developed practices to promote client involvement in services. Interestingly, the clients only contributed during one of the cooperation episodes.

#### 4.2.4 Summary of the results

To summarize, the analysis of the expansive turning points exemplified the mechanisms that indicated transitions from coordination to cooperation. The cooperation episodes enriched the discussion and added new aspects to it. In Object-orientation 1, they turned the discussion to the perspective of the client, and the participants discussed, for example, the importance of interaction, dialog and trust in service encounters. In Object-orientation 2, the focus of the discussion turned to professional practices and processes and their relation to the client involvement-based approach. The professionals also took part in the service development during these cooperation episodes. Thus, cooperation episodes could potentially construct a new client involvement-based object by enabling client involvement-based orientation in both the service encounters and the service system.

## 5. Discussion

The purpose of this study was to examine how clients and health and social care professionals co-developed client involvement ideas and constructed a new client involvement-based object during a developmental intervention. To create practices that involve health and social care clients in their own services and to offer them opportunities to develop these services alongside professionals (see, e.g. Nykänen *et al.*, 2022; Sihvo *et al.*, 2018), we arranged a Client Involvement Workshop in a Finnish social and welfare center. The analysis of the collective discussions demonstrated how a shared view of client involvement evolved and how a new client involvement-based object was constructed during the workshop. Next, we discuss these findings in more detail.

### 5.1 Building a shared view of client involvement

Our analysis of co-development revealed that the Client Involvement Workshop offered tools and an interactional space in which clients and professionals could analyze client involvement in a day-to-day setting, at the level of local activity (see also Osborne, 2018). During the intervention, the clients and professionals co-developed ideas and thematized client involvement through two interconnected object-orientations. Object-orientation 1, *Enabling client involvement in service encounters*, focused on the service of an individual client. The client–professional interaction, hearing the clients voice and enabling the clients to take the role of co-producer of their own services were predominant in the discussions (see also Viksveen *et al.*, 2022; Yang *et al.*, 2019). Object-orientation 2, *Promoting client involvement in the service system*, focused on how organizational practices and structures could enable client involvement and a smooth client experience, as well as ways in which to involve clients in service development. From the perspective of object construction, both orientations seemed to be central. Moving toward client involvement-based activity requires both tools and professional practices that support client involvement in service encounters and a focus on the service processes, structures, management and collaboration practices that enable clients to also be involved in the development of services (see also Engeström and Pyörälä, 2021). Thus, building client involvement-based activity and ensuring good-quality service processes requires finding a balance between the two orientations.

### 5.2 Client involvement-based object constructed through interaction

We found that in our data, coordination was the predominant mode of interaction during the Client Involvement Workshop; the object of discussions was only partially shared and each participant concentrated on their own perspective and organizational role. As known, collective object creation and the expansion acquired depends on the quality of interaction among the participants (Engeström, 2008; Fichtner, 1984). For developing new, in our case, client involvement-based services for frequent users, the coordination mode of interaction was not enough. A genuine dialog on shared problems was required, as well as going beyond the current organizational procedures and roles, thus moving toward cooperation or reflexive communication.

When facilitating co-development processes that aim to create something new, it is essential to understand the mechanisms behind the transitions between the modes of interaction. Our analysis showed that the clients played a central role in the expansive turning points (Kärkkäinen, 1999; Toiviainen, 2003) from coordination to cooperation: when they described their opinions and experiences and asked the professionals critical questions, it helped the participants direct the discussion from the viewpoint of the organization and organizational processes to that of the clients. As Fichtner (1984) has stated, cooperation is an essential condition for development and the most important instrument for practitioners, clients and professionals to generate a new quality of their services. During the cooperation episodes, the customary organization-based processes and practices were questioned and two object-orientations: *Enabling client involvement in service encounters* and *Promoting client involvement in the service system*, developed qualitatively. Thus, the substantial change in these orientations, and moving toward the client involvement-based object, were directly dependent on cooperation (Fichtner, 1984).

We found that clients play a meaningful role in co-development (also Boaz *et al.*, 2016). In Object-orientation 1, *Enabling client involvement in service encounters*, the clients not only initiated the cooperation episodes but they also played a central role in interaction. By bringing in their experiences from service encounters, they helped the professionals recognize the current challenges, and this helped co-develop ways in which to overcome these challenges. In Object-orientation 2, *Promoting client involvement in the service system,* the clients played a smaller role and only contributed to interaction during one cooperation episode. It may be that the clients were not as aware of the service processes and the service system as the professionals and thus felt that contributing was challenging.

We also observed that reflexive communication (Fichtner, 1984; Engeström, 2008) was not achieved during the workshop. The reason for this might be that in our case, the professionals who participated in the workshops came from different services and had not shared clients or collaboration practices. Moreover, the clients' experiences that were shared in the workshops were limited to only some of the services in question. However, crossing and reconceptualizing the assigned roles of services would be useful for developing client involvement-based services. This would need either a longer developmental process or a co-developer group of professionals and clients who deal with a shared object.

In our data, the majority of the participants in each session were professionals and dominated the discussion. Nevertheless, when the clients contributed to the discussion, their input was often relevant to the progression of the discussion (both the content and the social form of interaction). The experimental knowledge that the clients shared led to insights and priorities that would not otherwise have occurred (also Boaz *et al.*, 2016). Thus, to increase the client perspective in development, it might be worth finding a better balance between the number of clients and professional participants. It is also important that the interventionist ensures the equal participation of clients and professionals through multi-voiced discussions and listening to the voice of the client. This requires understanding and negotiating the power relations between clients and professionals (Nykänen *et al.*, 2022; Weiste *et al.*, 2020), as well as finding ways in which to support confidence within the actors.

### 5.3 Methodological comments

New client involvement-based activity cannot be created through mere discussion: It requires implementing and consolidating the expanded ideas in day-to-day work. The new concepts and ideas were tried out in practice through developmental experiments between the third and the fourth workshop sessions. The evaluation of these experiments during the fourth session indicated changes in activity. The value of the Client Involvement Workshop process was that it initiated collective learning and transformation toward a client involvement-based object. In this study, our analysis focused on the discussion and mode of interaction (Fichtner, 1984; Engeström, 2008) during the co-development of client involvement. The implementation of new ideas or concepts through co-development is not a linear process; it comprises tensions and potentials for expansion (Vetoshkina and Toiviainen, 2022). Moreover, deeper understanding of the changes, also in the long term, requires data and analysis of day-to-day work activity, service encounters and processes.

The data of our study came from a very speciﬁc context of organizational development: a Client Involvement Workshop in a Finnish municipal social and welfare center, and thus, our findings should be interpreted in relation to the historical development of that context. Thus, neither the object-orientations through which the client involvement-based object was constructed, nor the concrete manifestations implicating the modes of interaction or the turning points indicating the transition can be uncritically applied or transferred to other similar contexts.

As stated earlier, the number of participants in workshops was imbalanced, as we had several professionals but only two clients in our data. These clients had prior experience of participating in organizational development activities and obviously did not represent the highly heterogeneous group of general clients of the welfare center. However, they did have experience in being in the client position and were subsequently also able to voice their concerns and participate in the professional-driven development of organizational practices (see Aronson, 1993). The small number of clients in the workshops might have given the professionals dominance in the interaction (see Stevanovic *et al.*, 2022). The majority of the speaking turns in our data were indeed taken by the professionals, who guided the discussions in the small groups. Interestingly, however, the rare speaking turns of the clients still initiated transitions from coordination to cooperation. In future co-development processes, it would be worth discussing the clients' roles and the expectations concerning this at the beginning of the intervention. In this process, as interventionists, we allowed the clients to position themselves as they wished and did not strongly direct their role.

### 5.4 Implications for practice and further research

In terms of improving client involvement in health and social care services, this study highlights the importance of the processes in which clients and professionals co-develop new ideas and ways in which to implement these ideas as services. Moving toward client involvement-based practices requires remodeling both the client’s role and professional practices. This requires joint discussions among clients and professionals. As everyday life entails very few arenas for such discussions, Client Involvement Workshops fulfilled an important function by providing one such arena. In addition to fostering joint discussion between clients and professionals on client involvement, our study also highlights the importance of providing clients genuine possibilities for participation.

The clients should not be involved in development of services just to fulfill the obligation of involving clients. Rather, it is important to establish equal levels of participation. Keeping this in mind, in future development processes the number of clients and professionals should be well balanced. We also need more research on which developmental tools and working methods support equal participation and the clients’ involvement in the co-development processes.

## 6. Conclusions

The idea and principles of client involvement have been implemented in health and social care services for years, but real changes in day-to-day work are still ongoing. Client involvement is a value in itself, and organizations are being encouraged to involve clients in their own services and in service development. This requires promoting developmental activities, which in turn help construct new client involvement-based practices and regenerate the roles of both professionals and clients. This article analyzed Activity theory-based workshops, which offered an interactional space in which clients and professionals could co-develop client involvement and construct a new client involvement-based object. The collective object creation and expansion were analyzed on the basis of the contents of the discussions and by interpreting the discussions using the modes of interaction (Fichtner, 1984). The analysis showed that the client’s perspective and contributions were crucial for transitions from the coordination to the cooperation mode of interaction. This suggests that, in addition to being a central value in its own right, client involvement in the development workshops is also beneficial for professionals. It forces them to break across their professional boundaries and build a shared understanding of how client involvement could, and should, be realized in the development of services.

## Figures and Tables

**Table 1:** Modes of interaction

Mode of interaction	Object	Script
Coordination	Each participant/actor has a diverse object, or the objects are only partially shared	The script is based on organizational structures or tacitly assumed traditional roles. Each participant concentrates on their own service or assigned actions
Cooperation	Participants begin a collective object-construction by focusing on a shared problem and trying to find mutually acceptable ways to conceptualize and solve it	Participants go beyond the given script and assign roles, but do not explicitly question or reconceptualize them
Reflexive communication	Participants construct and reconceptualize a shared object	Participants reconceptualize their own roles, as well as the roles of different services and the interaction between actors

**Source(s):** Fichtner, 1984; Engeström, 2008

**Table 2:** Object-orientations and topics of discussion; clients’ and employees’ contributions and key expressions in each topic

Object-orientation	Topic of discussion	Episodes (N)	Episodes with clients’ contributions (N)	Clients who contributed (N/2)	Employees who contributed (N/20)	Key expressions
1. Enabling client involvement in service encounters	*Clients’ needs*	*17*	*10*	*2*	*14*	*“Individual needs”; “Client defines”; “Service for clients needs”*
	*Encounter (client and professional)*	*22*	*17*	*2*	*15*	*“Being heard”; “Being along”; “Trust”*
	*Setting a goal*	*3*	*-*	*-*	*2*	*“Personal goal”*
	*Planning the service*	*13*	*5*	*2*	*13*	*“Affecting the solutions”; “Being responsible”; “Being equal”*
	*Putting the service into practice*	*17*	*7*	*2*	*15*	*’Taking part’; “Controlling the own situation”; “Together with client”; “Supporting client”*
	*Decision-making*	*16*	*5*	*2*	*14*	*“Making the own decisions”; “Being willing to collaborate”; “Knowledge for decision* *-* *making”*
	*Collaboration of services*	*25*	*10*	*1*	*19*	*“Networking”; “Consultancy”; “Possibilities” “Empty words”*
	*Total*	*113*	*54*	*2*	*20*	
2. Promoting the client involvement in service system	*Managerial and organizational practices*	*9*	*-*	*-*	*8*	*“Involvement strategy”; “Scorecard”; “Involvement group”; “Resources for involvement work”*
	*Service structures*	*22*	*3*	*1*	*16*	*“Adequate services”; “Low-threshold services”; “Inter-professional collaboration”; “Limited resources”*
	*Service development*	*31*	*7*	*2*	*14*	*“Inviting clients' to take part”; “Handling customer feedback”; “Change agents”*
	*Total*	*62*	*10*	*2*	*17*	
TOTAL		*175*	*64*	*2*	*20*	

**Source(s):** Authors’ work

## Notes

1. *Act on the Status and Rights of Patients 785/1992* (Finland). https://www.finlex.fi/en/laki/kaannokset/1992/en19920785?search%5Btype%5D=pika&search%5Bkieli%5D%5B0%5D=en&search%5Bpika%5D=1992%2F785 (accessed 10 October 2021)

2. *Act on the Status and Rights of Social Welfare Clients 812/2000* (Finland). Retrieved from https://www.finlex.fi/en/laki/kaannokset/2000/en20000812?search%5Btype%5D=pika&search%5Bkieli%5D%5B0%5D=en&search%5Bpika%5D=2000%2F812 (accessed 10 October 2021)
